# Crystal structure of disodium dicobalt(II) iron(III) tris­(orthophosphate) with an alluaudite-like structure

**DOI:** 10.1107/S2056989015007926

**Published:** 2015-04-25

**Authors:** Adam Bouraima, Abderrazzak Assani, Mohamed Saadi, Thomas Makani, Lahcen El Ammari

**Affiliations:** a Laboratoire de Chimie du Solide Appliquée, Faculté des Sciences, Université Mohammed V, Avenue Ibn Battouta, BP 1014, Rabat, Morocco; b Département de chimie, Faculté des Sciences, Université des Sciences et Techniques de Masuku, BP 943, Franceville, Gabon

**Keywords:** crystal structure, transition metal phosphates, solid-state reaction synthesis, Na_2_Co_2_Fe(PO_4_)_3_, alluaudite-like structure

## Abstract

The title compound, Na_2_Co_2_Fe(PO_4_)_3_, was synthesized by a solid-state reaction. This new stoichiometric phase crystallizes in an alluaudite-like structure. In this structure, all atoms are in general positions except for four atoms which are located at the special positions of the *C*2/*c* space group. One Co atom, one P and one Na atom are all located on Wyckoff position 4*e* (2), while the second Na atom is located on an inversion centre 4*a* (-1). The other Co and Fe atoms occupy a general position with a statistical distribution. The open framework results from [(Co,Fe)_2_O_10_] units of edge-sharing [(Co,Fe)O_6_] octa­hedra, which alternate with [CoO_6_] octa­hedra that form infinite chains running along the [10-1] direction. These chains are linked together through PO_4_ tetra­hedra by the sharing of vertices so as to build layers perpendicular to [010]. The three-dimensional framework is accomplished by the stacking of these layers, leading to the formation of two types of tunnels parallel to [010] in which the Na^+^ cations are located, each cation being surrounded by eight O atoms.

## Abstract 2

The transition metal orthophosphates Na_2_ *M*_2_Fe(PO_4_)_3_ (*M* = Co, Ni) crystallize in an alluaudite-type structure. The chains characterizing the alluaudite structure are then built up from [*M*_2_O_10_] units alternating with [FeO_6_] octa­hedra.

## Chemical context   

A particular focus of ours concerns compounds with alluaudite-type structures, and we set the objective of synthesising new transition-metal-based phosphates within the well-known alluaudite family. We are inter­ested in this because transition-metal phosphates are of great inter­est with applications in several fields. Compounds belonging to the large structural family of derivatives (Moore, 1971 ▸) have been of continuing inter­est due to their structural properties, such as their open-framework architecture and their physical properties. Moreover, the flexibility of the alluaudite structure will, no doubt, permit the use of alluaudite-type phosphates for practical applications, such as corrosion inhibition, passivation of metal surfaces and catalysis (Korzenski *et al.*, 1999 ▸). These materials abound in magnetic properties of metallic phosphate. Transition metals can play an important role in microporous skeletons by supplying an active catalytic site keeping the selectivity of frames (Weil *et al.*, 2009 ▸). Metallic phosphates present a multitude of structural wealth which are the object of studies of catalysts (Viter & Nagornyi, 2009 ▸; Gao & Gao, 2005 ▸), ion exchange (Clearfield, 1988 ▸) and the positive electrode in lithium and sodium batteries (Trad *et al.*, 2010 ▸). As a result of the presence of channels parallel to [100], alluaudite-type compounds exhibit electronic and/or ionic conductivity, as has been shown by Warner *et al.* (1993 ▸). In this context, we have explored *A*_2_O–*M*O–P_2_O_5_ systems, where *A* is a monovalent cation and *M* a divalent cation. A new alluaudite structure of formula Na_2_Co_2_Fe(PO_4_)_3_ was synthesized by solid-state reaction. During our investigation of these systems, we characterized the following compounds: AgMg_3_(PO_4_)(HPO_4_)_2_ (Assani *et al.*, 2011*a* ▸), Ag_2_Ni_3_(HPO_4_)(PO_4_)_2_ (Assani *et al.*, 2011*b* ▸) and Na_2_Ni_2_Fe(PO_4_)_3_ (Essehli *et al.*, 2011 ▸). The present paper reports the solid-state synthesis and characterization of a new transition-metal phosphate, namely, Na_2_Co_2_Fe(PO_4_)_3_.

## Structural commentary   

In the refinement of the first model of this structure, we placed the Fe atom in Wyckoff position 4*e* and Co in the general position 8*f*. The results of the refinement of this model are acceptable if we disregard the high weight values. However, bond-valence-sum calculations (Brown & Altermatt, 1985 ▸) are not in favor of this model and, consequently, the examination of all possible models led to the best one in which half of the Co, Na, and P atoms are in Wyckoff position 4*e*, and the second Na atom is in position 4*a* of the *C*2/*c* space group, the remaining Co and Fe fulfilling the 8*f* site. In this case, bond-valence-sum calculations for Co2^2+^, Co1^2+^, Fe1^2+^, Na1^+^, Na2^+^, P1^5+^ and P2^5+^ ions are as expected, *viz* 1.78, 2.02, 2.81, 1.25, 0.94, 4.98 and 4.99 valence units, respectively.

**Figure 1:**
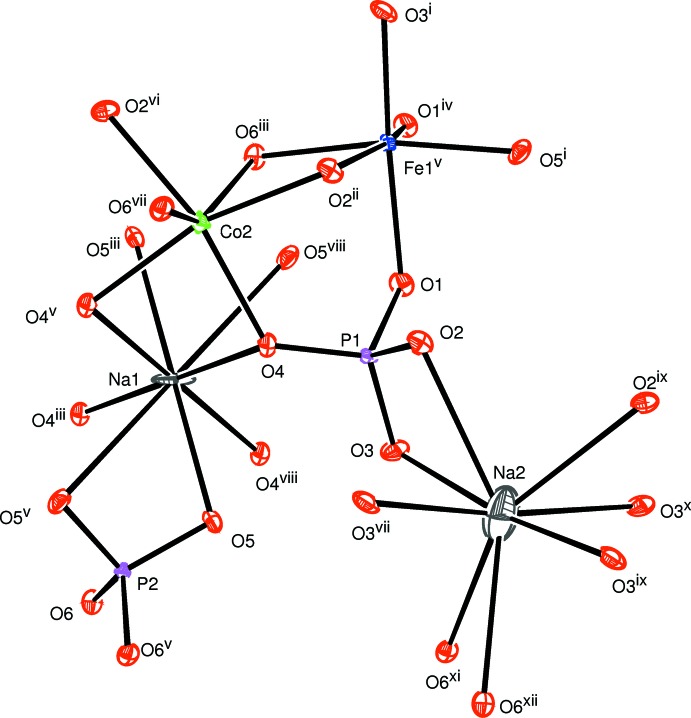
The principal building units in the structure of the title compound. Displacement ellipsoids are drawn at the 50% probability level. Symmetry codes: (i) −*x* + 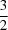, *y* + 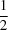, −*z* + 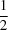; (ii) −*x* + 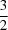, −*y* + 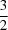, −*z* + 1; (iii) −*x* + 1, −*y* + 1, −*z*; (iv) −*x* + 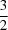, −*y* + 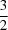, −*z*; (v) −*x* + 1, *y*, −*z* + 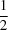; (vi) *x* − 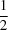, −*y* + 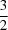, *z* − 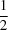; (vii) *x*, −*y* + 1, *z* + 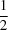; (viii) *x*, −*y* + 1, *z* − 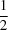; (ix) −*x* + 2, *y*, −*z* + 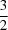; (x) −*x* + 2, −*y* + 1, −*z* + 1; (xi) *x* + 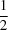, −*y* + 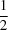, *z* + 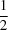; (xii) −*x* + 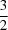, −*y* + 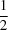, −*z* + 1.

**Figure 2:**
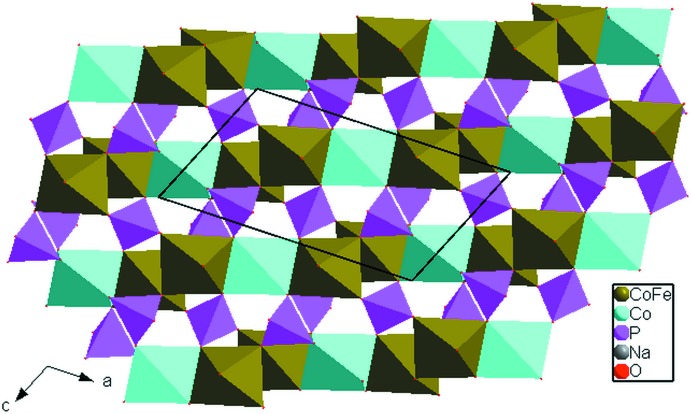
A layer perpendicular to the *b* axis, resulting from the chains connected *via* the vertices of the PO_4_ tetra­hedra.

**Figure 3:**
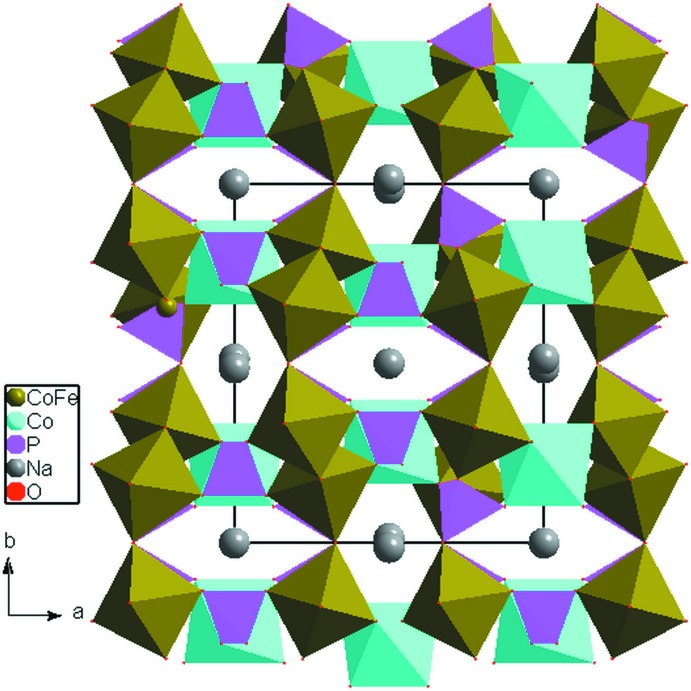
Polyhedral representation of Na_2_Co_2_Fe(PO_4_)_3_ showing the tunnels running along the [001] direction.

The new phase of formula Na_2_Co_2_Fe(PO_4_)_3_ crystallizes in the alluaudite type. The structure of this compound is built up from two edge-sharing [(Co,Fe)O_6_] octa­hedra, leading to the formation of [(Co,Fe)_2_O_10_] dimers that are connected by a common edge to [CoO_6_] octa­hedra, as shown in Fig. 1 ▸. The linkage of alternating [CoO_6_] and [(Co,Fe)_2_O_10_] octa­hedra leads to infinite chains along the [10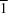] direction. These chains held together *via* the vertices of the PO_4_ tetra­hedra in such a way as to build layers perpendicular to [010] (Fig. 2 ▸). The junction of different octa­hedra by common vertices of PO_4_ tetra­hedra form an open three-dimensional framework that delimits two types of tunnels parallel to [100] and [001] accommodating the Na^+^ cations, as shown in Fig. 3 ▸. In the tunnels, each sodium atom is surrounded by eight oxygen atoms with Na1—O and Na2—O bond lengths varying between 2.2895 (9) and 2.8754 (10) Å) and between 2.3940 (9) and 2.8513 (16) Å, respectively.

## Synthesis and crystallization   

Na_2_Co_2_Fe(PO_4_)_3_ was synthesized by a solid-state reaction by mixing the precursors of sodium (Na_2_CO_3_), cobalt (CoCO_3_), iron (Fe_2_O_3_) and phospho­ric acid 85% wt. The various precursors were taken in the molar ratio Na:Co:Fe:P = 2:2:1:3.

After different heat treatments in a platinum crucible up to 873 K, the reaction mixture was heated to the melting point of 1000 K. The molten product was then cooled to room temperature at a rate of 5 K h^−1^. The resulting product contained brown crystals of a suitable size for the X-ray diffraction study.

## Refinement   

Crystal data, data collection and structure refinement details are summarized in Table 1 ▸. The same *x*, *y* and *z* parameters and anisotropic displacement parameters are used for Co1 and Fe1 sharing the same site. Three reflections, (042), (110) and (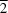
42), probably affected by the beam-stop, were removed during the last refinement cycle. The highest peak and the deepest hole in the final Fourier map are at 0.40 Å and 0.42 Å from Na1 and Na2, respectively.

## Supplementary Material

Crystal structure: contains datablock(s) I. DOI: 10.1107/S2056989015007926/br2248sup1.cif

Structure factors: contains datablock(s) I. DOI: 10.1107/S2056989015007926/br2248Isup2.hkl

CCDC reference: 1060932

Additional supporting information:  crystallographic information; 3D view; checkCIF report

## Figures and Tables

**Table 1:** Experimental details

Crystal data	
Chemical formula	Na_2_Co_2_Fe(PO_4_)_3_
*M*_r_	504.60
Crystal system, space group	Monoclinic, *C*2/*c*
Temperature (K)	296
*a*, *b*, *c* ()	11.7106(6), 12.4083(7), 6.4285(3)
()	113.959(2)
*V* (^3^)	853.63(8)
*Z*	4
Radiation type	Mo *K*
(mm^1^)	6.26
Crystal size (mm)	0.31 0.25 0.19

Data collection	
Diffractometer	Bruker X8 APEX
Absorption correction	Multi-scan (*SADABS*; Bruker, 2009 ▸)
*T*_min_, *T*_max_	0.504, 0.748
No. of measured, independent and observed [*I* > 2(*I*)] reflections	15289, 1882, 1807
*R*_int_	0.030
(sin /)_max_ (^1^)	0.806

Refinement	
*R*[*F* ^2^ > 2(*F* ^2^)], *wR*(*F* ^2^), *S*	0.016, 0.046, 1.10
No. of reflections	1879
No. of parameters	95
_max_, _min_ (e ^3^)	0.70, 0.92

Computer programs: *APEX2* and *SAINT* (Bruker, 2009 ▸), *SHELXS97* and *SHELXL97* (Sheldrick, 2008 ▸), *ORTEP-3 for Windows* (Farrugia, 2012 ▸), *DIAMOND* (Brandenburg, 2006 ▸) and *publCIF* (Westrip, 2010 ▸).

## supplementary crystallographic information

### Crystal data

**Table:**

Na_2_Co_2_Fe(PO_4_)_3_	*F*(000) = 972
*M**_r_* = 504.60	*D*_x_ = 3.926 Mg m^−^^3^
Monoclinic, *C*2/*c*	Mo *K*α radiation, λ = 0.71073 Å
Hall symbol: -c 2yc	Cell parameters from 1882 reflections
*a* = 11.7106 (6) Å	θ = 2.5–34.9°
*b* = 12.4083 (7) Å	µ = 6.26 mm^−^^1^
*c* = 6.4285 (3) Å	*T* = 296 K
β = 113.959 (2)°	Block, brown
*V* = 853.63 (8) Å^3^	0.31 × 0.25 × 0.19 mm
*Z* = 4	

### Data collection

**Table:**

Bruker X8 APEX diffractometer	1882 independent reflections
Radiation source: fine-focus sealed tube	1807 reflections with *I* > 2σ(*I*)
Graphite monochromator	*R*_int_ = 0.030
φ and ω scans	θ_max_ = 34.9°, θ_min_ = 2.5°
Absorption correction: multi-scan (*SADABS*; Bruker, 2009)	*h* = −18→18
*T*_min_ = 0.504, *T*_max_ = 0.748	*k* = −20→20
15289 measured reflections	*l* = −9→10

### Refinement

**Table:**

Refinement on *F*^2^	Primary atom site location: structure-invariant direct methods
Least-squares matrix: full	Secondary atom site location: difference Fourier map
*R*[*F*^2^ > 2σ(*F*^2^)] = 0.016	*w* = 1/[σ^2^(*F*_o_^2^) + (0.026*P*)^2^ + 1.033*P*] where *P* = (*F*_o_^2^ + 2*F*_c_^2^)/3
*wR*(*F*^2^) = 0.046	(Δ/σ)_max_ = 0.002
*S* = 1.10	Δρ_max_ = 0.70 e Å^−^^3^
1879 reflections	Δρ_min_ = −0.92 e Å^−^^3^
95 parameters	Extinction correction: *SHELXL97* (Sheldrick, 2008), Fc^*^=kFc[1+0.001xFc^2^λ^3^/sin(2θ)]^-1/4^
0 restraints	Extinction coefficient: 0.0049 (3)

### Special details

**Table:**

Geometry. All esds (except the esd in the dihedral angle between two l.s. planes) are estimated using the full covariance matrix. The cell esds are taken into account individually in the estimation of esds in distances, angles and torsion angles; correlations between esds in cell parameters are only used when they are defined by crystal symmetry. An approximate (isotropic) treatment of cell esds is used for estimating esds involving l.s. planes.
Refinement. Refinement of *F*^2^ against all reflections. The weighted *R*-factor *wR* and goodness of fit *S* are based on *F*^2^, conventional *R*-factors *R* are based on *F*, with *F* set to zero for negative *F*^2^. The threshold expression of *F*^2^ > σ(*F*^2^) is used only for calculating *R*-factors(gt) *etc*. and is not relevant to the choice of reflections for refinement. *R*-factors based on *F*^2^ are statistically about twice as large as those based on *F*, and *R*- factors based on all data will be even larger.

### Fractional atomic coordinates and isotropic or equivalent isotropic displacement parameters (Å^2^)

**Table:**

	*x*	*y*	*z*	*U*_iso_*/*U*_eq_	Occ. (<1)
Fe1	0.717910 (14)	0.842242 (12)	0.13094 (2)	0.00487 (5)	0.50
Co1	0.717910 (14)	0.842242 (12)	0.13094 (2)	0.00487 (5)	0.50
Co2	0.5000	0.731171 (18)	0.2500	0.00645 (5)	
P1	0.76483 (3)	0.60996 (2)	0.37608 (4)	0.00406 (6)	
P2	0.5000	0.29046 (3)	0.2500	0.00393 (7)	
Na1	0.5000	0.5000	0.0000	0.01623 (17)	
Na2	1.0000	0.48710 (12)	0.7500	0.0343 (3)	
O1	0.77862 (8)	0.67773 (7)	0.18650 (14)	0.00805 (14)	
O2	0.83828 (8)	0.66574 (7)	0.60820 (14)	0.00755 (14)	
O3	0.82670 (9)	0.49990 (7)	0.38761 (16)	0.00954 (15)	
O4	0.62676 (8)	0.60150 (7)	0.32737 (15)	0.00871 (14)	
O5	0.60276 (8)	0.36657 (7)	0.25279 (15)	0.00938 (15)	
O6	0.45930 (8)	0.21880 (7)	0.03503 (13)	0.00683 (13)	

### Atomic displacement parameters (Å^2^)

**Table:**

	*U*^11^	*U*^22^	*U*^33^	*U*^12^	*U*^13^	*U*^23^
Fe1	0.00449 (7)	0.00506 (7)	0.00514 (7)	−0.00034 (4)	0.00203 (5)	−0.00037 (4)
Co1	0.00449 (7)	0.00506 (7)	0.00514 (7)	−0.00034 (4)	0.00203 (5)	−0.00037 (4)
Co2	0.00659 (9)	0.00646 (9)	0.00728 (9)	0.000	0.00381 (7)	0.000
P1	0.00506 (11)	0.00343 (11)	0.00348 (10)	−0.00004 (8)	0.00150 (8)	−0.00003 (7)
P2	0.00357 (15)	0.00423 (15)	0.00352 (14)	0.000	0.00094 (11)	0.000
Na1	0.0262 (4)	0.0032 (3)	0.0068 (3)	0.0017 (3)	−0.0061 (3)	0.0008 (2)
Na2	0.0187 (5)	0.0569 (8)	0.0218 (5)	0.000	0.0026 (4)	0.000
O1	0.0094 (3)	0.0097 (3)	0.0052 (3)	−0.0011 (3)	0.0031 (3)	0.0014 (3)
O2	0.0097 (3)	0.0073 (3)	0.0049 (3)	−0.0018 (3)	0.0021 (3)	−0.0015 (2)
O3	0.0113 (4)	0.0061 (3)	0.0109 (3)	0.0026 (3)	0.0043 (3)	−0.0012 (3)
O4	0.0057 (3)	0.0080 (3)	0.0124 (3)	−0.0002 (3)	0.0037 (3)	0.0002 (3)
O5	0.0063 (3)	0.0081 (3)	0.0124 (3)	−0.0022 (3)	0.0024 (3)	0.0024 (3)
O6	0.0065 (3)	0.0089 (3)	0.0047 (3)	−0.0008 (3)	0.0019 (2)	−0.0019 (2)

### Geometric parameters (Å, º)

**Table:**

Fe1—O5^i^	1.9456 (9)	P2—O6^v^	1.5468 (8)
Fe1—O3^i^	2.0158 (9)	P2—O6	1.5468 (8)
Fe1—O2^ii^	2.0374 (9)	Na1—O5^iii^	2.2895 (9)
Fe1—O6^iii^	2.0543 (9)	Na1—O5	2.2895 (9)
Fe1—O1^iv^	2.0724 (8)	Na1—O4^iii^	2.3835 (9)
Fe1—O1	2.1434 (9)	Na1—O4	2.3836 (9)
Co2—O4^v^	2.1072 (9)	Na1—O4^viii^	2.5217 (9)
Co2—O4	2.1072 (9)	Na1—O4^v^	2.5218 (9)
Co2—O2^ii^	2.1567 (9)	Na1—O5^v^	2.8754 (10)
Co2—O2^vi^	2.1568 (9)	Na1—O5^viii^	2.8754 (10)
Co2—O6^vii^	2.1632 (8)	Na2—O3^ix^	2.3940 (9)
Co2—O6^iii^	2.1632 (8)	Na2—O3	2.3940 (9)
P1—O4	1.5206 (9)	Na2—O3^x^	2.5269 (10)
P1—O3	1.5336 (9)	Na2—O3^vii^	2.5269 (10)
P1—O1	1.5422 (9)	Na2—O2^ix^	2.8158 (15)
P1—O2	1.5502 (9)	Na2—O2	2.8158 (15)
P2—O5^v^	1.5240 (9)	Na2—O6^xi^	2.8513 (16)
P2—O5	1.5241 (9)	Na2—O6^xii^	2.8513 (16)
			
O5^i^—Fe1—O3^i^	94.91 (4)	O5—Na1—O4^viii^	73.59 (3)
O5^i^—Fe1—O2^ii^	110.51 (4)	O4^iii^—Na1—O4^viii^	67.32 (4)
O3^i^—Fe1—O2^ii^	86.18 (4)	O4—Na1—O4^viii^	112.68 (4)
O5^i^—Fe1—O6^iii^	164.12 (4)	O5^iii^—Na1—O4^v^	73.59 (3)
O3^i^—Fe1—O6^iii^	98.29 (4)	O5—Na1—O4^v^	106.41 (3)
O2^ii^—Fe1—O6^iii^	79.27 (3)	O4^iii^—Na1—O4^v^	112.68 (4)
O5^i^—Fe1—O1^iv^	86.97 (4)	O4—Na1—O4^v^	67.32 (4)
O3^i^—Fe1—O1^iv^	99.56 (4)	O4^viii^—Na1—O4^v^	180.0
O2^ii^—Fe1—O1^iv^	161.21 (4)	O5^iii^—Na1—O5^v^	126.25 (4)
O6^iii^—Fe1—O1^iv^	82.19 (3)	O5—Na1—O5^v^	53.75 (4)
O5^i^—Fe1—O1	81.40 (4)	O4^iii^—Na1—O5^v^	86.18 (3)
O3^i^—Fe1—O1	174.04 (4)	O4—Na1—O5^v^	93.82 (3)
O2^ii^—Fe1—O1	90.73 (3)	O4^viii^—Na1—O5^v^	114.14 (3)
O6^iii^—Fe1—O1	86.11 (3)	O4^v^—Na1—O5^v^	65.86 (3)
O1^iv^—Fe1—O1	84.97 (3)	O5^iii^—Na1—O5^viii^	53.75 (4)
O4^v^—Co2—O4	80.44 (5)	O5—Na1—O5^viii^	126.25 (4)
O4^v^—Co2—O2^ii^	165.05 (3)	O4^iii^—Na1—O5^viii^	93.82 (3)
O4—Co2—O2^ii^	86.54 (3)	O4—Na1—O5^viii^	86.18 (3)
O4^v^—Co2—O2^vi^	86.54 (3)	O4^viii^—Na1—O5^viii^	65.86 (3)
O4—Co2—O2^vi^	165.05 (3)	O4^v^—Na1—O5^viii^	114.14 (3)
O2^ii^—Co2—O2^vi^	107.25 (5)	O5^v^—Na1—O5^viii^	180.0
O4^v^—Co2—O6^vii^	92.44 (3)	O3^ix^—Na2—O3	172.39 (8)
O4—Co2—O6^vii^	113.30 (3)	O3^ix^—Na2—O3^x^	81.52 (3)
O2^ii^—Co2—O6^vii^	85.96 (3)	O3—Na2—O3^x^	97.99 (3)
O2^vi^—Co2—O6^vii^	74.34 (3)	O3^ix^—Na2—O3^vii^	97.99 (3)
O4^v^—Co2—O6^iii^	113.30 (3)	O3—Na2—O3^vii^	81.52 (3)
O4—Co2—O6^iii^	92.44 (3)	O3^x^—Na2—O3^vii^	172.68 (8)
O2^ii^—Co2—O6^iii^	74.34 (3)	O3^ix^—Na2—O2^ix^	55.93 (3)
O2^vi^—Co2—O6^iii^	85.96 (3)	O3—Na2—O2^ix^	117.11 (5)
O6^vii^—Co2—O6^iii^	146.65 (5)	O3^x^—Na2—O2^ix^	62.15 (3)
O4—P1—O3	112.98 (5)	O3^vii^—Na2—O2^ix^	111.51 (5)
O4—P1—O1	108.59 (5)	O3^ix^—Na2—O2	117.11 (5)
O3—P1—O1	108.95 (5)	O3—Na2—O2	55.93 (3)
O4—P1—O2	110.92 (5)	O3^x^—Na2—O2	111.51 (5)
O3—P1—O2	106.53 (5)	O3^vii^—Na2—O2	62.15 (3)
O1—P1—O2	108.78 (5)	O2^ix^—Na2—O2	76.15 (5)
O5^v^—P2—O5	103.42 (7)	O3^ix^—Na2—O6^xi^	116.10 (5)
O5^v^—P2—O6^v^	108.79 (5)	O3—Na2—O6^xi^	71.28 (4)
O5—P2—O6^v^	112.97 (5)	O3^x^—Na2—O6^xi^	83.54 (4)
O5^v^—P2—O6	112.97 (5)	O3^vii^—Na2—O6^xi^	103.11 (4)
O5—P2—O6	108.79 (5)	O2^ix^—Na2—O6^xi^	145.12 (3)
O6^v^—P2—O6	109.82 (7)	O2—Na2—O6^xi^	126.19 (2)
O5^iii^—Na1—O5	180.0	O3^ix^—Na2—O6^xii^	71.28 (4)
O5^iii^—Na1—O4^iii^	78.23 (3)	O3—Na2—O6^xii^	116.10 (5)
O5—Na1—O4^iii^	101.77 (3)	O3^x^—Na2—O6^xii^	103.11 (4)
O5^iii^—Na1—O4	101.77 (3)	O3^vii^—Na2—O6^xii^	83.54 (4)
O5—Na1—O4	78.23 (3)	O2^ix^—Na2—O6^xii^	126.19 (2)
O4^iii^—Na1—O4	180.0	O2—Na2—O6^xii^	145.12 (3)
O5^iii^—Na1—O4^viii^	106.41 (3)	O6^xi^—Na2—O6^xii^	52.71 (4)

Symmetry codes: (i) −*x*+3/2, *y*+1/2, −*z*+1/2; (ii) −*x*+3/2, −*y*+3/2, −*z*+1; (iii) −*x*+1, −*y*+1, −*z*; (iv) −*x*+3/2, −*y*+3/2, −*z*; (v) −*x*+1, *y*, −*z*+1/2; (vi) *x*−1/2, −*y*+3/2, *z*−1/2; (vii) *x*, −*y*+1, *z*+1/2; (viii) *x*, −*y*+1, *z*−1/2; (ix) −*x*+2, *y*, −*z*+3/2; (x) −*x*+2, −*y*+1, −*z*+1; (xi) *x*+1/2, −*y*+1/2, *z*+1/2; (xii) −*x*+3/2, −*y*+1/2, −*z*+1.
